# Supplementary material for: Atomic layer deposition for core-shell microparticle vaccines enabling programmable antigen delivery to lymph nodes enhance humoral immune responses
Source: bioRxiv. 2026 Jun 2:2026.05.29.728600. Preprint. [Version 1] doi: 10.64898/2026.05.29.728600 (PMC13252159; doi:10.64898/2026.05.29.728600)
Supplement: Supplement 1 [file media-1.docx]

**Supplementary Information for:**

**Atomic layer deposition for core-shell microparticle vaccines enabling programmable antigen delivery to lymph nodes enhance humoral immune responses**

Namit Chaudhary^1,2^, Holly J. Coleman^3^, Elias V. Paolone^1^, Jiancheng Yu^1^, Amir W. Ledbetter^4^, Ashley A. Lemnios^1^, Agnes A. Walsh^1^, Heikyung Suh^1^, Alexander Wang^1^, Maryam M. Mansoor^5^, JoLynn B. Giancola^1^, Mariane B. Melo^1,2^, Amber M. Rauch^3^, Erika S. Langsfeld^6^, Urvi R. Parlikar^3^, Marisa O. Pacheco^3^, Carly A. Williams^3^, Hans H. Funke^3^, Robert L. Garcea^6^, Theodore W. Randolph^3^, Darrell J. Irvine^1,2,7^

1. Department of Immunology and Microbiology, The Scripps Research Institute, La Jolla, CA 92037
2. Howard Hughes Medical Institute, Chevy Chase, MD 20815
3. Department of Chemical and Biological Engineering, University of Colorado, Boulder, CO 80309
4. Koch Institute for Integrative Cancer Research, Massachusetts Institute of Technology, Cambridge, MA 02139
5. Department of Bioengineering, University of California San Diego, La Jolla, CA 92093
6. The BioFrontiers Institute, University of Colorado, Boulder, CO 80309
7. Center for HIV/AIDS Vaccine Immunology and Immunogen Discovery (Scripps CHAVD), The Scripps Research Institute, La Jolla, CA 92037

Corresponding authors: Darrell J. Irvine (djirvine@scripps.edu), Theodore W. Randolph (theodore.randolph@colorado.edu)

**Supplementary Table 1. Spray drying formulations evaluated for HIV Env trimer encapsulation.**

| **Formulation** | **Trehalose (wt%)** | **Histidine (mM)** | **Tween 20 (wt%)** | **Hydroxyethylsulfate (wt%)** | **pH** |
| --- | --- | --- | --- | --- | --- |
| F1 | 9.5 | 50 | 0.2 | 2.5 | 6.5 |
| F2 | 9.5 | 50 | 0.2 | 0 | 6.5 |
| F3 | 9.5 | 50 | 0 | 2.5 | 6.5 |
| F4 | 9.5 | 50 | 0 | 0 | 6.5 |
| F5 | 9.5 | 50 | 0.2 | 2.5 | 5.5 |
| F6 | 9.5 | 50 | 0.2 | 0 | 5.5 |
| F7 | 9.5 | 50 | 0 | 2.5 | 5.5 |
| F8 | 9.5 | 50 | 0 | 0 | 5.5 |
| F9 | 9.5 | 10 | 0.2 | 2.5 | 6.5 |
| F10 | 9.5 | 10 | 0.2 | 0 | 6.5 |
| F11 | 9.5 | 10 | 0 | 2.5 | 6.5 |
| F12 | 9.5 | 10 | 0 | 0 | 6.5 |
| F13 | 9.5 | 10 | 0.2 | 2.5 | 5.5 |
| F14 | 9.5 | 10 | 0.2 | 0 | 5.5 |
| F15 | 9.5 | 10 | 0 | 2.5 | 5.5 |
| F16 | 9.5 | 10 | 0 | 0 | 5.5 |

All samples contained 50 µg/mL N332-GT2 trimer.
