# Supplementary figures and images for: Atomic layer deposition for core-shell microparticle vaccines enabling programmable antigen delivery to lymph nodes enhance humoral immune responses
Source: bioRxiv. 2026 Jun 2:2026.05.29.728600. Preprint. [Version 1] doi: 10.64898/2026.05.29.728600 (PMC13252159; doi:10.64898/2026.05.29.728600)

Extended Data Figure 1

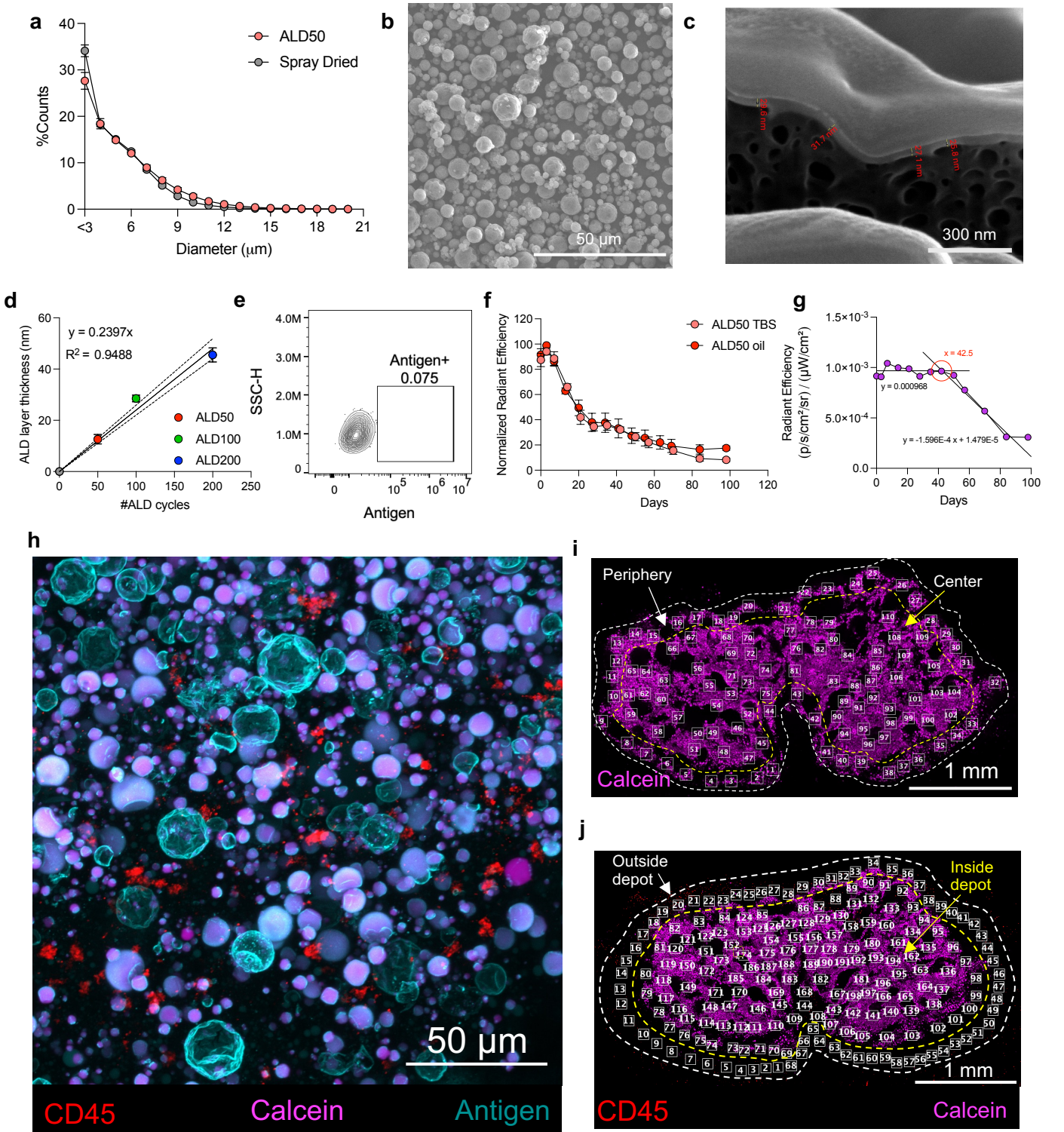

Extended Data Figure 2

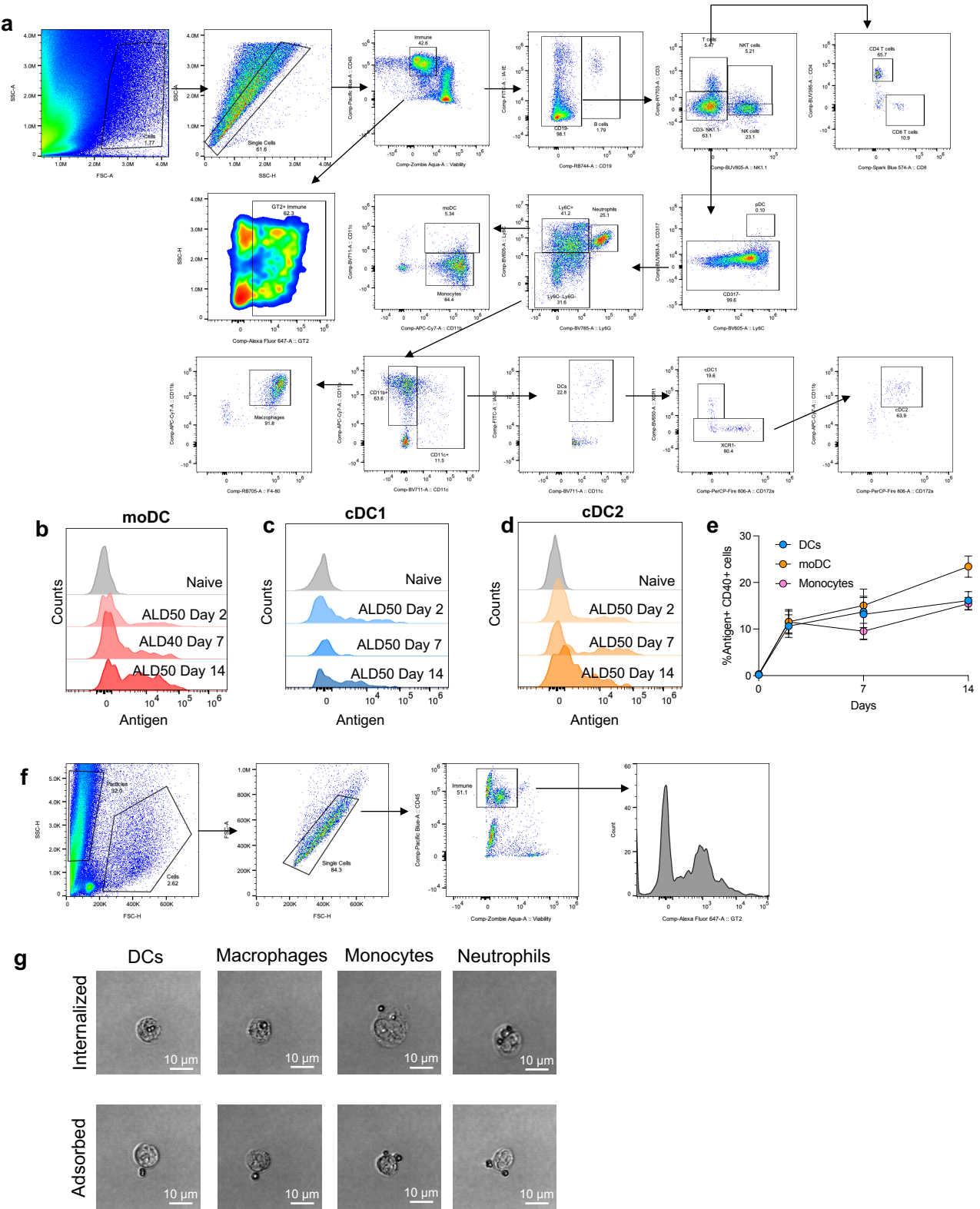

Extended Data Figure 3

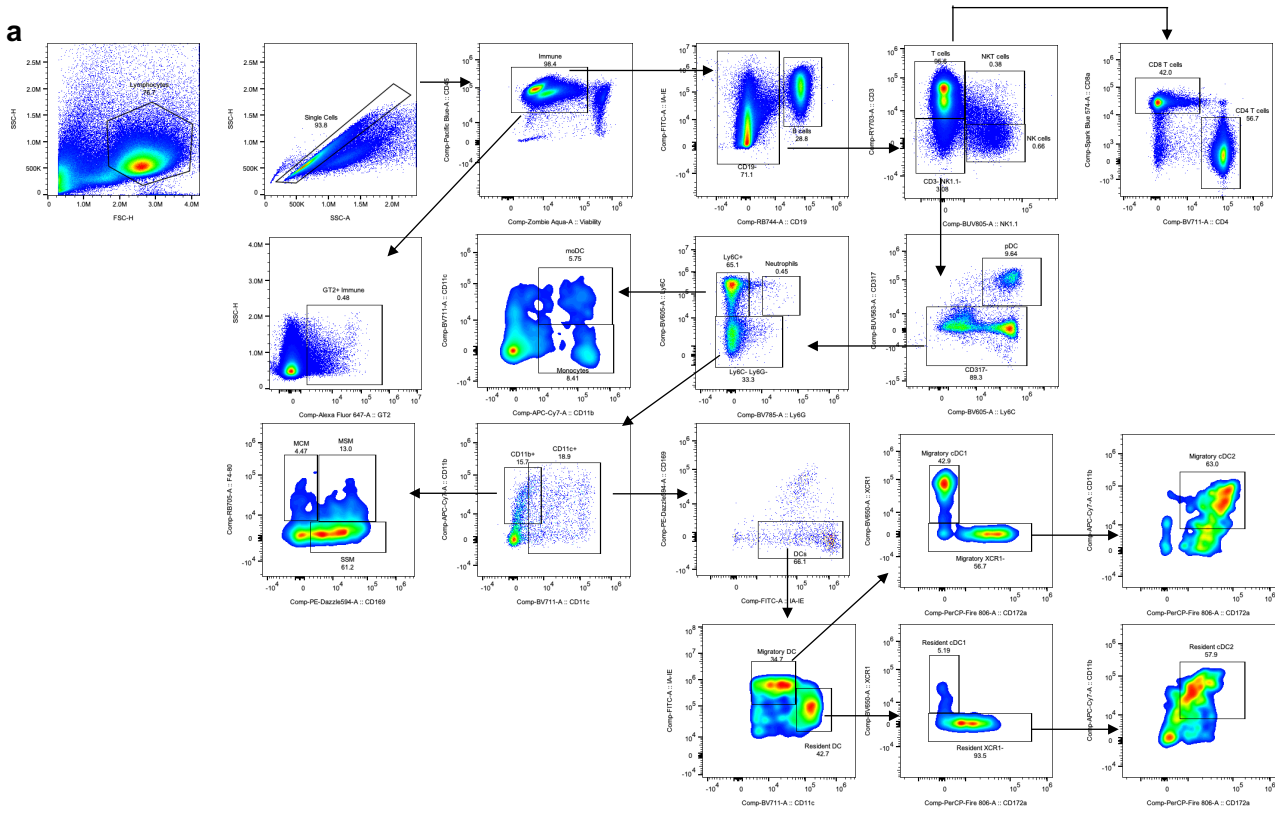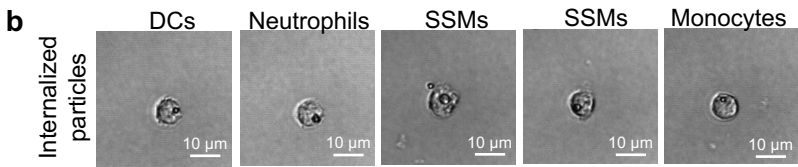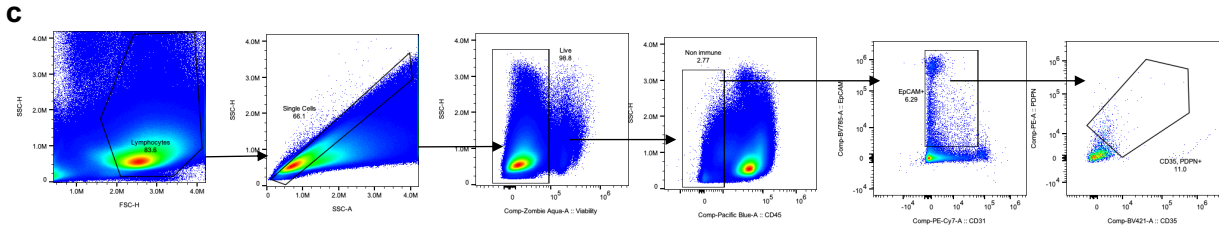

Extended Data Figure 4

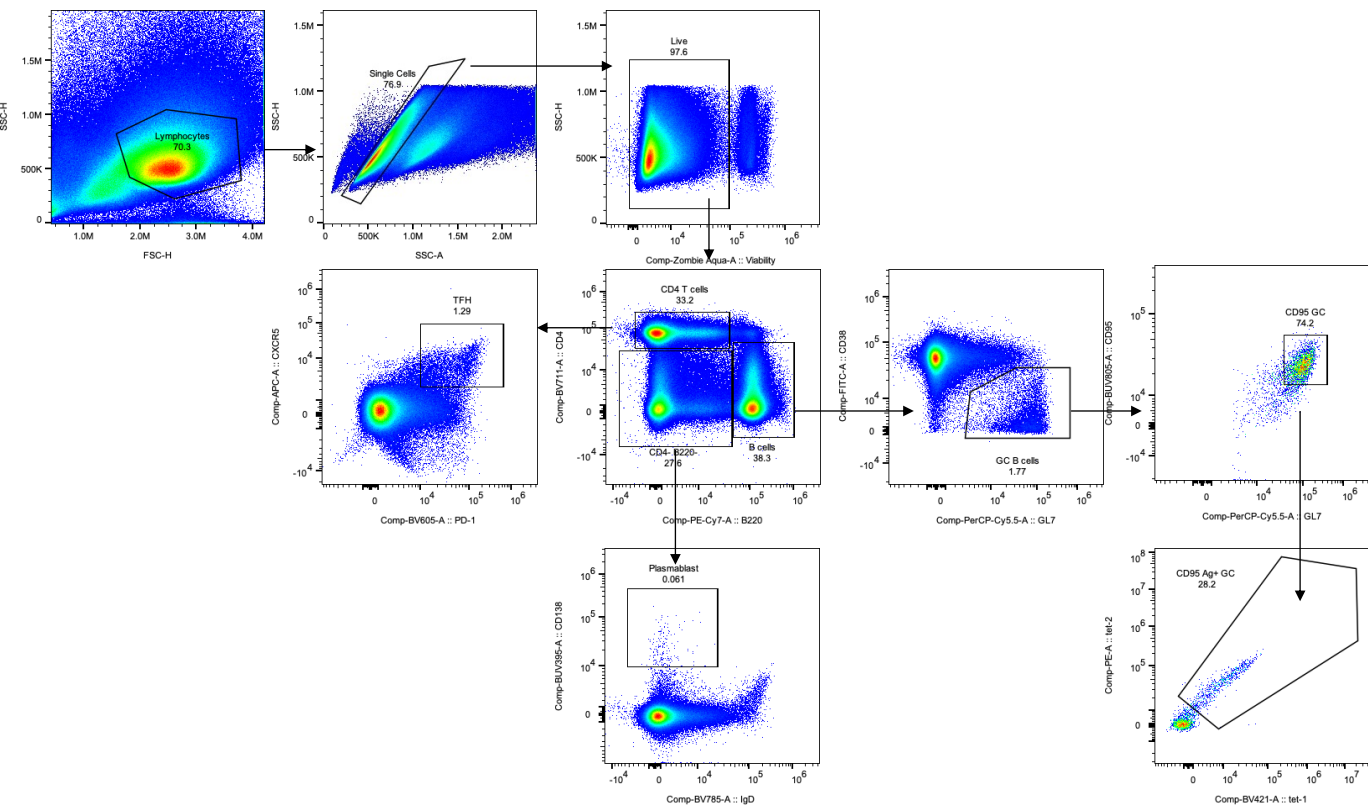

Supplement: Supplement 2 — Extended Data Fig. 1. Physical characterization of ALD microparticles and functional behavior at injection sites. a) Antigen was spray-dried using formulation 1 and coated with 50 layers of alumina. Particle size distributions were determined for spray-dried and ALD microparticles by flow imaging microscopy. b) Morphology of spray-dried particles was visualized via scanning electron microscopy. c-d) Thickness of alumina layers deposited by ALD on microparticles was determined by FIB-SEM. Shown are a representative FIB-SEM image of the alumina shell in ALD100 particles (c) and measured alumina shell thickness as a function ALD cycle number (d). Each ALD cycle deposited 0.24±0.02 nm alumina, as determined from a linear plot (r2=0.95) of alumina layer thickness vs. number of ALD cycles. e) BMDCs (n = 4 samples/group) were incubated with 5 μg/mL free Alexa Fluor 647-labeled antigen for 24 hr, followed by flow cytometry analysis. Shown is representative flow cytometry plot of antigen uptake. f) Albino C57BL/6 mice (n = 5 animals/group) were injected s.c. with ALD50 particles encapsulating 10 μg Alexa Fluor 647–labeled antigen suspended in either triglyceride oil or Tris-buffered saline. Antigen persistence at the injection site was monitored longitudinally by whole-animal fluorescence imaging and quantified as normalized radiant efficiency over time. g) Animals were immunized with ALD particles encapsulating fluorescent antigen as in Fig. 3b and antigen fluorescence at the injection site was tracked over time. To estimate the time point when the bulk of antigen clearance begins, the initial plateau and subsequent decay regions of the fluorescence data were fit by linear regression, and the time point for intersection of the two best fit lines was taken as the time of release initiation. h-j) C57BL/6 mice (n = 4 animals/group) were immunized with 5 μg Alexa Fluor 647-labeled antigen and 7.5 μg calcein administered as ALD particles suspended in oil. Injection sites were ex [file media-2.pdf]
